# Supplementary material for: Serotonin and dopamine modulate aging in response to food odor and availability
Source: Nat Commun. 2022 Jun 7;13:3271. doi: 10.1038/s41467-022-30869-5 (PMC9174215; doi:10.1038/s41467-022-30869-5)
Supplement: Supplementary file 1 — Supplementary Information [file 41467_2022_30869_MOESM1_ESM.docx]

**SUPPLEMENTARY INFORMATION**

**Serotonin and dopamine modulate aging in response to food odor and availability**

**
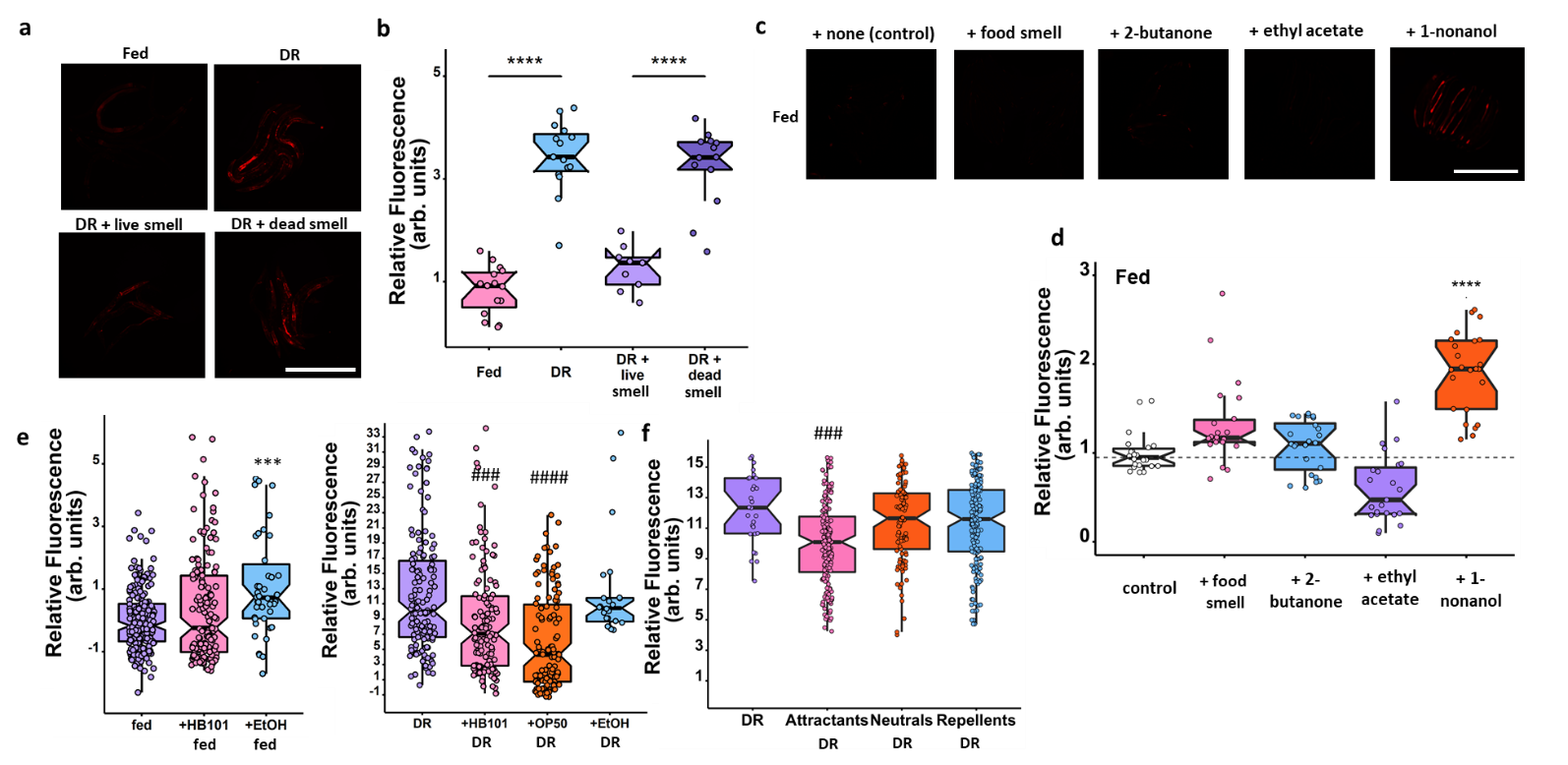
**

**
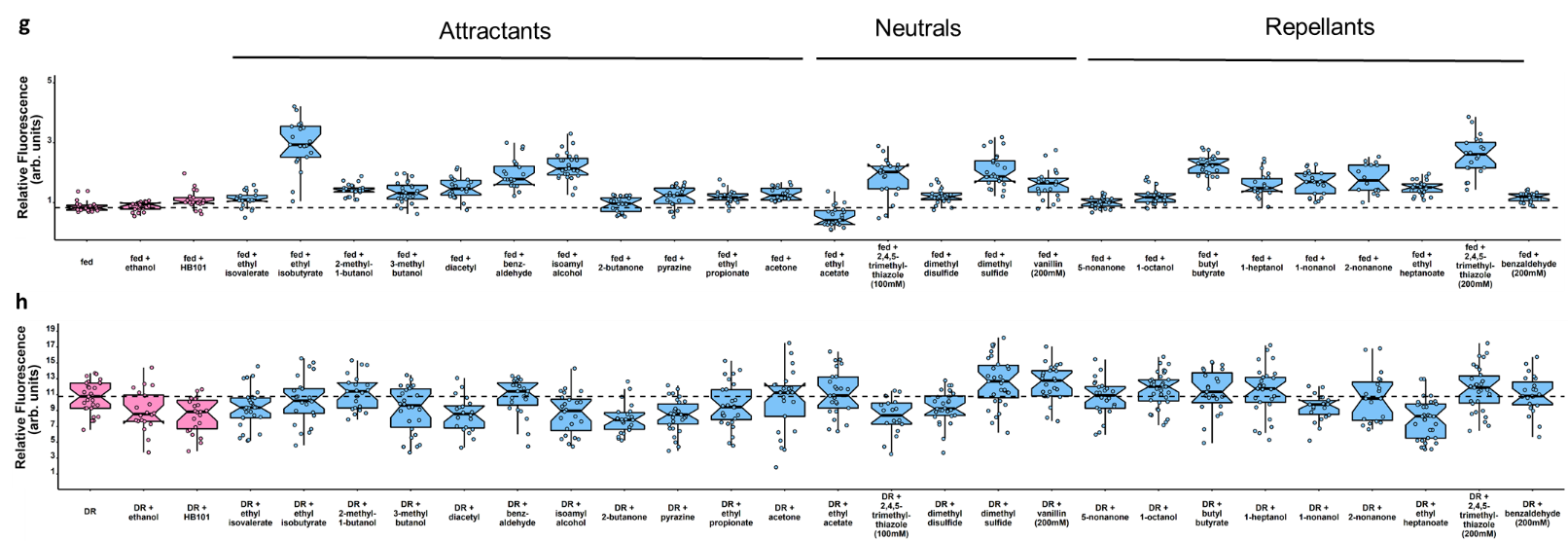
**

**
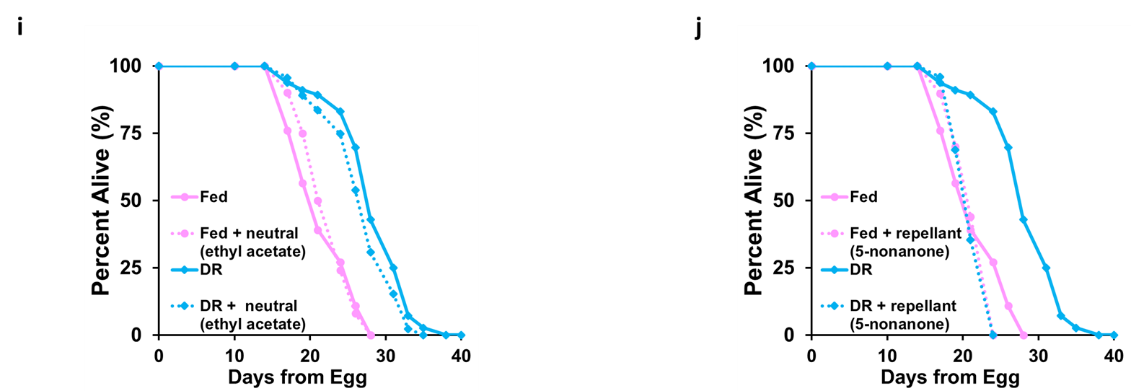
**

**Supplementary Fig. 1. Odorant effects on *fmo-2* expression.** Images (**a**) and quantification (**b**) of individual *fmo-2p::mCherry* worms on fed (pink), DR (blue), and smell with live OP50 (light purple) or PFA-killed OP50 (dark purple). Scale bar, 1 mm. n = 15 (fed), 15 (DR), 9 (DR + live smell), 14 (DR + dead smell) biologically independent animals. p-value = 6.84e-12 (DR vs. fed), 7.66e-08 (DR + live smell vs. DR + dead smell). **** denotes P<.0001 (Welch Two Sample t-test, two-sided). Additional images (**c**) and quantification (**d**) of individual *fmo-2p::mCherry* worms on fed plates exposed to food smell (pink) or attractive (2-butanone in blue), neutral (ethyl acetate in purple), or repellant (1-nonanol in orange) odorants. Scale bar, 1 mm. n = 22 (control), 22 (fed + food smell), 24 (fed + 2-butanone), 24 (fed + ethyl acetate), 24 (fed + 1-nonanol) biologically independent animals. p-value = 2.43e-10 (Fed vs fed + 1-nonanol). Summary of controls HB101 (pink), OP50 (orange) and ethanol (blue) effects on fed and DR conditions across experiments (**e**). n = 170 (fed), 123 (fed + HB101), 42 (fed + EtOH), 162 (DR), 136 (DR + HB101), 137 (DR + OP50), 20 (DR + EtOH) biologically independent animals. p-value = 0.000669 (Fed vs fed + ethanol), 0.00209 (DR vs DR + HB101 smell), 6.59e-08 (DR vs DR + OP50 smell). Summary of worms on DR treated with attractant (pink), neutral (orange) or repellant (blue) compounds compared to DR (**f**). n = 28 (DR), 179 (Attractants + DR), 129 (Neutrals+ DR), 188 (Repellents + DR) biologically independent animals. p-value = 0.00405 (DR vs DR + attractants). All odorant effects on fed (**g**) and DR (**h**) conditions. n = 22 (fed control), 22 (fed + ethanol), 21 (fed + HB101), 21 (fed + ethyl isovalerate), 21 (fed + ethyl isobutyrate), 22 (fed + 2-methyl-1-butanol), 24 (fed + 3-methyl butanol), 21 (fed + diacetyl), 20 (fed + benzaldehyde), 27 (fed + isoamyl alcohol), 24 (fed + 2-butanone), 22 (fed + pyrazine), 22 (fed + ethyl propionate), 22 (fed + acetone), 25 (fed + ethyl acetate), 21 (fed + 2, 4, 5-trimethyl-thiazole (100mM)), 23 (fed + dimethyl disulfide (100mM)), 25 (fed + dimethyl sulfide), 24 (fed + vanillin (200mM), 22 (fed + 5-nonanone), 23 (fed + 1-octanol), 26 (fed + butyl butyrate), 22 (fed + 1-heptanol), 24 (fed + 1-nonanol), 16 (fed + 2-nonanone), 24 (fed + ethyl heptanoate), 20 (fed + 2, 4, 5-trimethylthiazole (200mM)), 19 (fed + benzaldehyde (200mM)) biologically independent animals in **g.** n = 28 (DR control), 20 (DR + ethanol), 23 (DR + HB101), 29 (DR + ethyl isovalerate), 24 (DR + ethyl isobutyrate), 21 (DR + 2-methyl-1-butanol), 26 (DR + 3-methyl butanol), 20 (DR + diacetyl), 23 (DR + benzaldehyde), 24 (DR + isoamyl alcohol), 22 (DR + 2-butanone), 30 (DR + pyrazine), 28 (DR + ethyl propionate), 26 (DR + acetone), 25 (DR + ethyl acetate), 20 (DR + 2, 4, 5-trimethyl-thiazole (100mM)), 28 (DR + dimethyl disulfide (100mM)), 31 (DR + dimethyl sulfide), 25 (DR + vanillin (200mM), 24 (DR + 5-nonanone), 35 (DR + 1-octanol), 23 (DR + butyl butyrate), 29 (DR + 1-heptanol), 22 (DR + 1-nonanol), 19 (DR + 2-nonanone), 36 (DR + ethyl heptanoate), 30 (DR + 2, 4, 5-trimethylthiazole (200mM)), 23 (DR + benzaldehyde (200mM)) biologically independent animals in **h**. Survival curves of N2 (WT) animals fed (pink) or DR (blue) under normal conditions (solid lines) or subjected to attractive (**i**) repellant (**j**) odorants (dotted line). *** denotes P < 0.001 when compared to fed (Welch Two Sample t-test, two-sided). ### denotes P < 0.001 and #### denotes P<.0001 when compared to DR (Welch Two Sample t-test, two-sided). The box plots display the median by the middle line of the box. The upper boundary of the box indicates the 75% interquartile range, and the lower boundary indicates the 25% interquartile range.


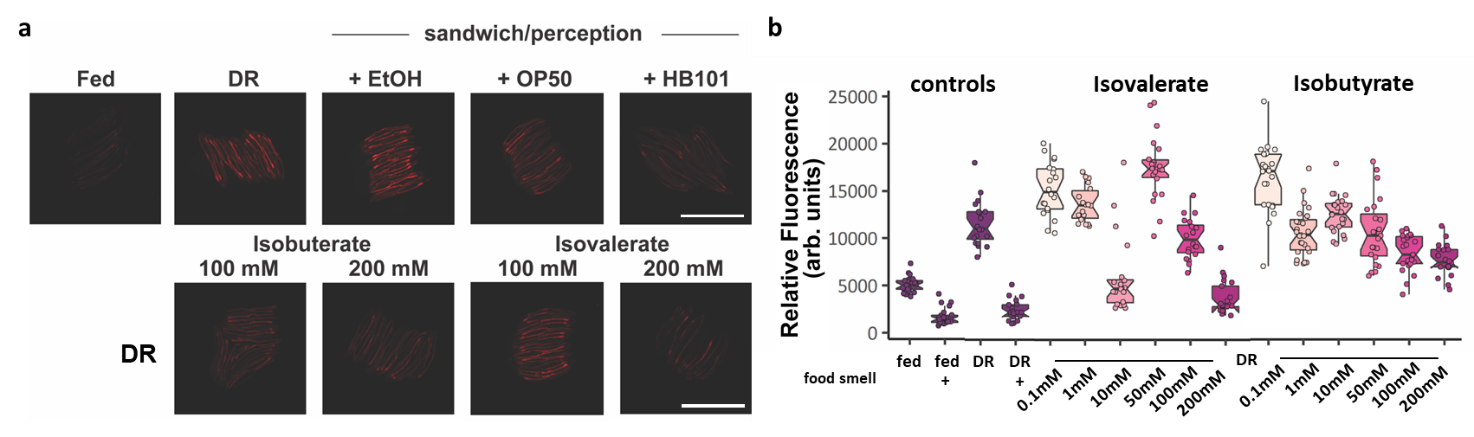


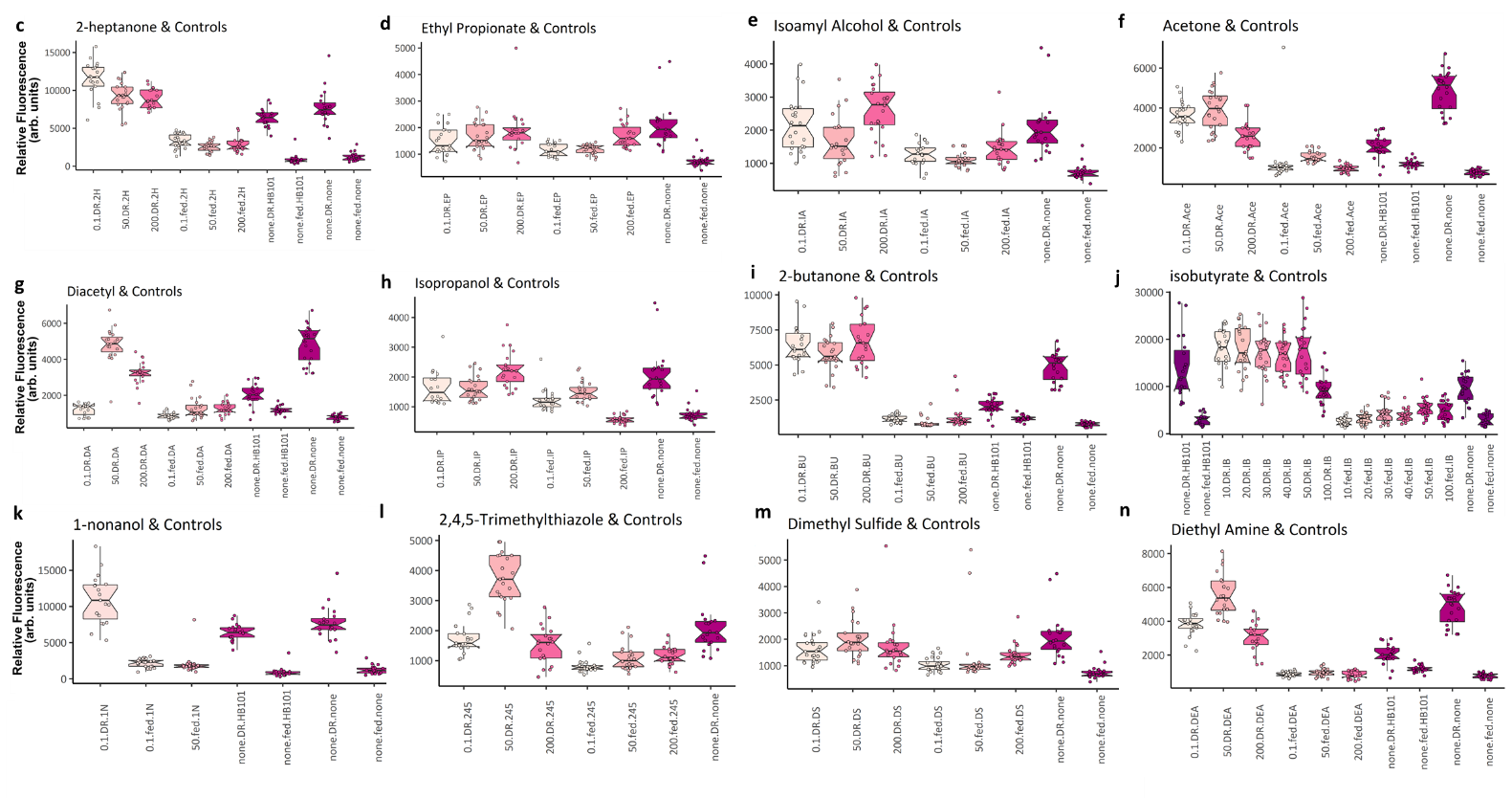


**
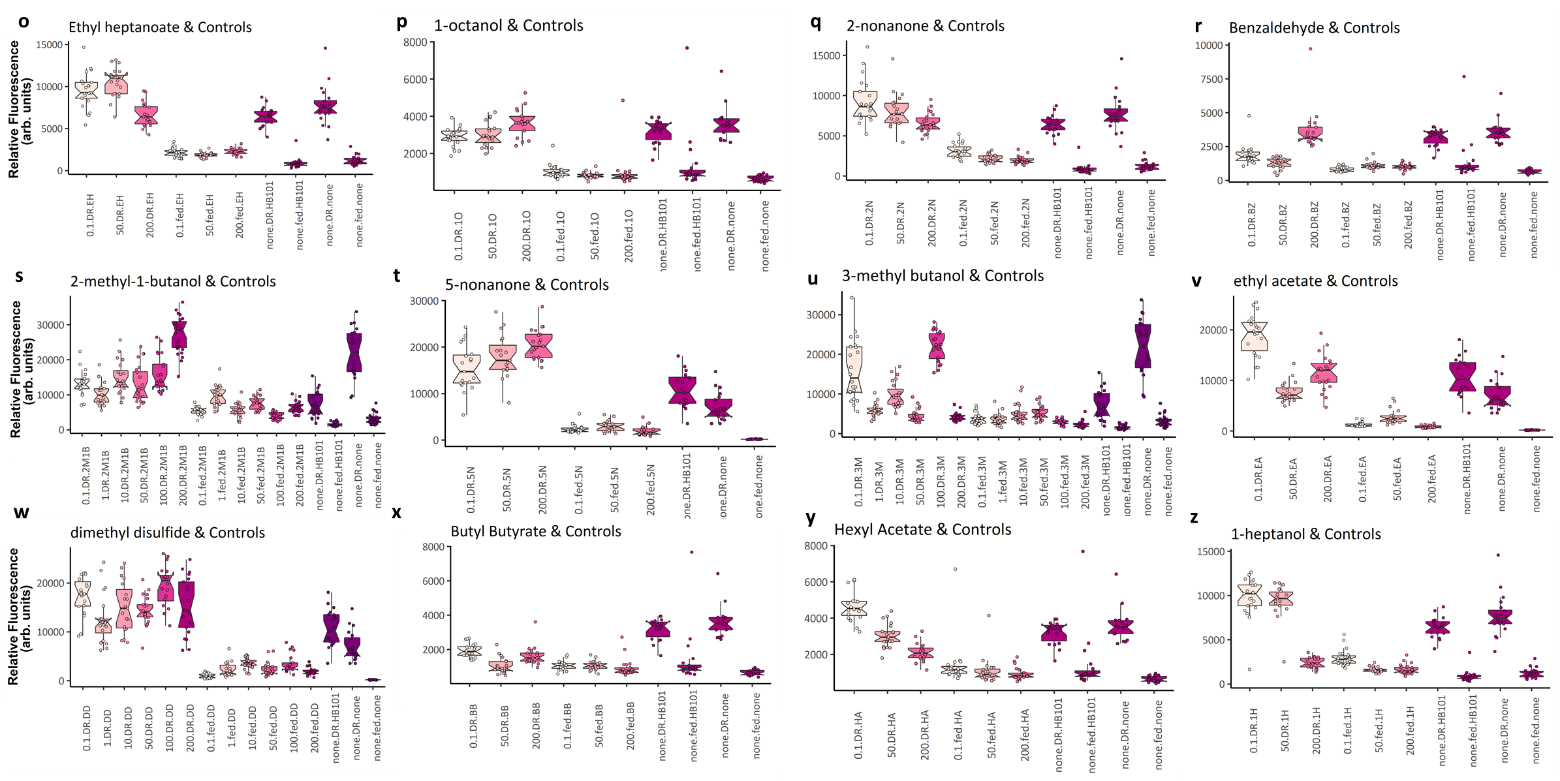
**

**Supplementary Fig. 2.** **Titration experiments of odorants tested**. Panels show representative images (**a**) and quantification of *fmo-2p::mCherry* under DR (b-z). Scale bar, 1 mm. n = 20 (controls fed), 20 (controls fed +food smell), 17 (controls DR), 18 (controls DR + food smell), 20 (DR + 0.1mM isovalerate), 18 (DR + 1mM isovalerate), 20 (DR + 10mM isovalerate), 22 (DR + 50mM isovalerate), 20 (DR + 100mM isovalerate), 21 (DR + 200mM isovalerate), 24 (DR + 0.1mM isobutyrate), 27 (DR + 1mM isobutyrate), 23 (DR + 10mM isobutyrate), 23 (DR + 50mM isobutyrate), 23 (DR + 100mM isobutyrate), 22 (DR + 200mM isobutyrate) biologically independent animals in **b**. n = 20 (0.1mM DR), 21 (50mM DR), 19 (200mM DR), 26 (0.1 mM fed), 22 (50 mM fed), 22 (200 mM fed), 19 (DR + HB101), 21 (fed + HB101), 20 (DR), 20 (fed) biologically independent animals in **c**. n = 21 (0.1mM DR), 23 (50mM DR), 20 (200mM DR), 19 (0.1mM fed), 23 (50mM fed), 22 (200mM fed), 21 (DR), 22 (fed) biologically independent animals in **d**. n = 25 (0.1mM DR), 22 (50mM DR), 24 (200mM DR), 21 (0.1mM fed), 21 (50mM fed), 22 (200mM fed), 21 (DR), 22 (fed) biologically independent animals in **e**. n = 23 (0.1mM DR), 24 (50mM DR), 21 (200mM DR), 22 (0.1mM fed), 19 (50mM fed), 21 (200mM fed), 21 (DR + HB101), 21 (fed + HB101), 24 (DR), 21 (fed) biologically independent animals in **f**. n = 19 (0.1mM DR), 19 (50mM DR), 22 (200mM DR), 20 (0.1mM fed), 21 (50mM fed), 20 (200mM fed), 21 (DR + HB101), 21 (fed + HB101), 24 (DR), 21 (fed) biologically independent animals in **g**. n = 16 (0.1mM DR), 24 (50mM DR), 21 (200mM DR), 20 (0.1mM fed), 21 (50mM fed), 23 (200mM fed), 21 (DR), 22 (fed) biologically independent animals in **h**. n = 22 (0.1mM DR), 21 (50mM DR), 19 (200mM DR), 21 (0.1mM fed), 21 (50mM fed), 22 (200mM fed), 21 (DR + HB101), 21 (fed + HB101), 24 (DR), 21 (fed) biologically independent animals in **i**. n = 19 (DR + HB101), 18 (fed + HB101), 20 (10mM DR), 20 (20mM DR), 23 (30mM DR), 21 (40mM DR), 22 (50mM DR), 20 (100mM DR), 21 (10mM fed), 22 (20mM fed), 23 (30mM fed), 22 (40mM fed), 22 (50mM fed), 21 (100mM fed), 20 (DR), 21 (fed) biologically independent animals in **j**. n = 19 (0.1mM DR), 21 (0.1mM fed), 22 (50mM fed), 19 (DR + HB101), 21 (fed + HB101), 20 (DR), 20 (fed) biologically independent animals in **k**. n = 19 (0.1mM DR), 21 (50mM DR), 20 (200mM DR), 21 (0.1mM fed), 20 (50mM fed), 21 (200mM fed), 21 (DR) biologically independent animals in **l**. n = 24 (0.1mM DR), 24 (50mM DR), 22 (200mM DR), 24 (0.1mM fed), 20 (50mM fed), 22 (200mM fed), 21 (DR), 22 (fed) biologically independent animals in **m**. n = 20 (0.1mM DR), 20 (50mM DR), 19 (200mM DR), 23 (0.1mM fed), 24 (50mM fed), 20 (200mM fed), 21 (DR + HB101), 21 (fed + HB101), 24 (DR), 21 (fed) biologically independent animals in **n**. n = 21 (0.1mM DR), 23 (50mM DR), 21 (200mM DR), 21 (0.1mM fed), 21 (50mM fed), 20 (200mM fed), 19 (DR + HB101), 21 (fed + HB101), 20 (DR), 20 (fed) biologically independent animals in **o**. n = 21 (0.1mM DR), 19 (50mM DR), 17 (200mM DR), 20 (0.1mM fed), 20 (50mM fed), 21 (200mM fed), 18 (DR + HB101), 20 (fed + HB101), 17 (DR), 20 (fed) biologically independent animals in **p**. n = 21 (0.1mM DR), 20 (50mM DR), 22 (200mM DR), 23 (0.1mM fed), 23 (50mM fed), 23 (200mM fed), 19 (DR + HB101), 21 (fed + HB101), 20 (DR), 20 (fed) biologically independent animals in **q**. n = 20 (0.1mM DR), 21 (50mM DR), 20 (200mM DR), 21 (0.1mM fed), 20 (50mM fed), 21 (200mM fed), 18 (DR + HB101), 20 (fed + HB101), 17 (DR), 20 (fed) biologically independent animals in **r**. n = 18 (0.1mM DR), 19 (1mM DR), 20 (10mM DR), 24 (50mM DR), 21 (100mM DR), 27 (200mM DR), 21 (0.1mM fed), 22 (1mM fed), 22 (10mM fed), 21 (50mM fed), 21 (100mM fed), 21 (200mM fed), 20 (DR + HB101), 21 (fed + HB101), 21 (DR), 23 (fed) biologically independent animals in **s**. n = 20 (0.1mM DR), 19 (50mM DR), 21 (200mM DR), 20 (0.1mM fed), 21 (50mM fed), 19 (200mM fed), 20 (DR), 19 (fed + HB101), 23 (fed) biologically independent animals in **t**. n = 22 (0.1mM DR), 19 (1mM DR), 23 (10mM DR), 21 (50mM DR), 21 (100mM DR), 19 (200mM DR), 21 (0.1mM fed), 23 (1mM fed), 23 (10mM fed), 21 (50mM fed), 20 (100mM fed), 24 (200mM fed), 20 (DR + HB101), 21 (fed + HB101), 21 (DR), 23 (fed) biologically independent animals in **u**. n = 22 (0.1mM DR), 21 (50mM DR), 21 (200mM DR), 21 (0.1mM fed), 20 (50mM fed), 20 (200mM fed), 20 (DR), 19 (fed + HB101), 23 (fed) biologically independent animals in **v**. n = 19 (0.1mM DR), 24 (1mM DR), 19 (10mM DR), 21 (50mM DR), 20 (100mM DR), 21 (200mM DR), 20 (0.1mM fed), 20 (1mM fed), 22 (10mM fed), 21 (50mM fed), 21 (100mM fed), 20 (200mM fed), 20 (DR), 19 (fed + HB101), 23 (fed) biologically independent animals in **w**. n = 20 (0.1mM DR), 22 (50mM DR), 21 (200mM DR), 20 (0.1mM fed), 20 (50mM fed), 21 (200mM fed), 18 (DR + HB101), 20 (fed + HB101), 17 (DR), 20 (fed) biologically independent animals in **x**. n = 19 (0.1mM DR), 19 (50mM DR), 19 (200mM DR), 20 (0.1mM fed), 20 (50mM fed), 24 (200mM fed), 18 (DR + HB101), 20 (fed + HB101), 17 (DR), 20 (fed) biologically independent animals in **y**. n = 21 (0.1mM DR), 18 (50mM DR), 21 (200mM DR), 23 (0.1mM fed), 22 (50mM fed), 24 (200mM fed), 19 (DR + HB101), 21 (fed + HB101), 20 (DR), 20 (fed) biologically independent animals in **z**. Dosing and preparation can be found in Supplementary Table 3. The box plots display the median by the middle line of the box. The upper boundary of the box indicates the 75% interquartile range, and the lower boundary indicates the 25% interquartile range.

**
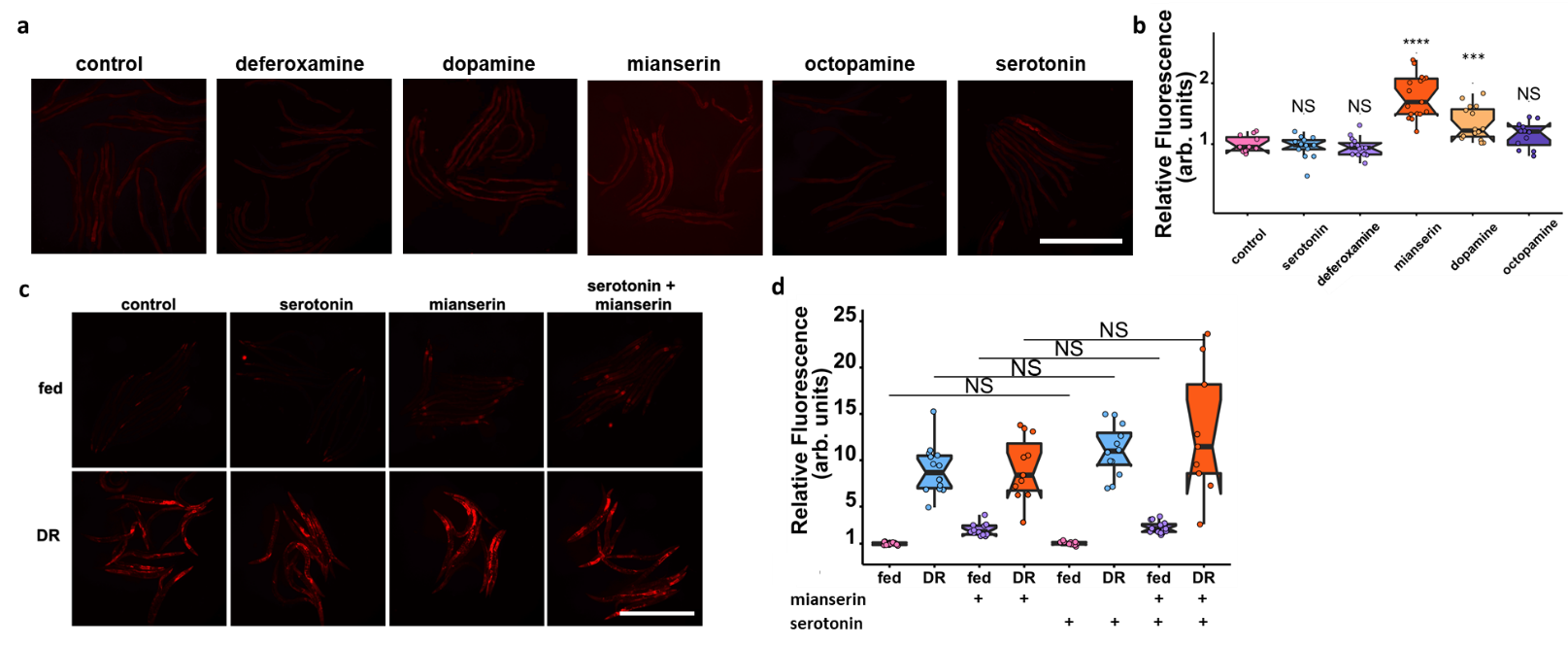
**

**
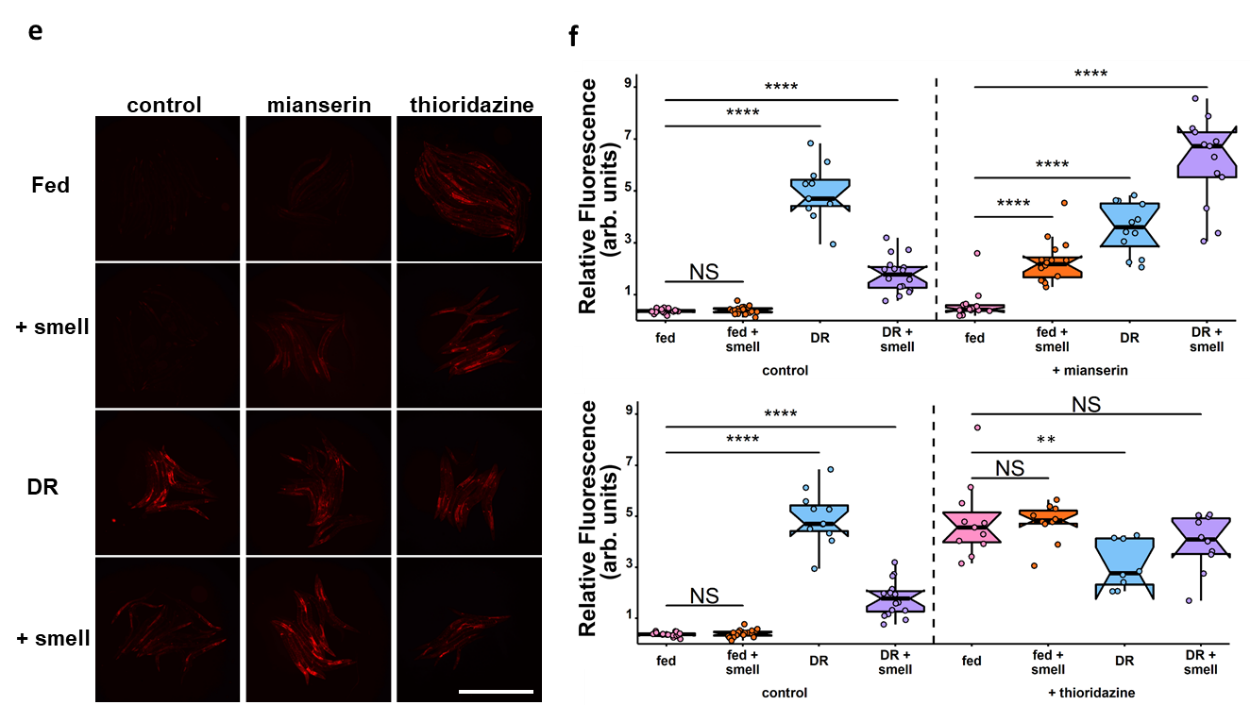

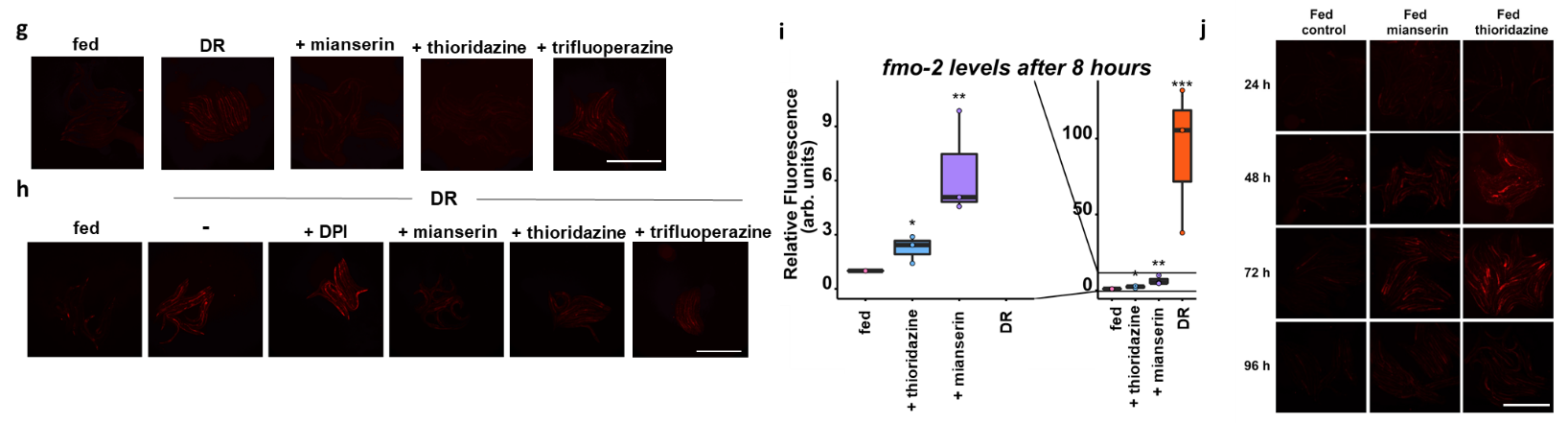
**

**
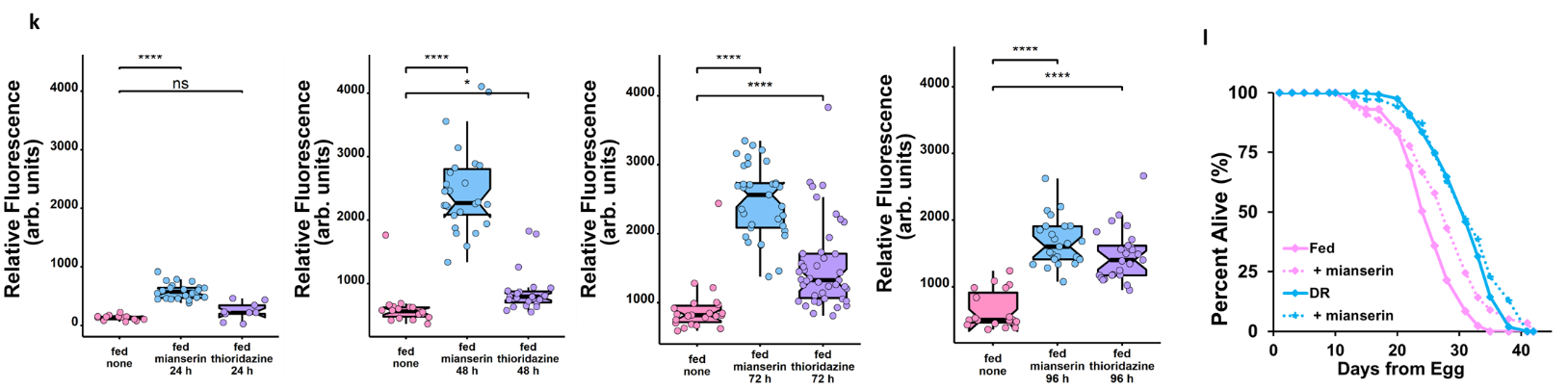
**

**Supplementary Fig. 3**. **Induction of *fmo-2* by neuromodulators.** Images (**a**) and quantification (**b**) of *fmo-2p*::*mCherry* worms exposed to water (pink), serotonin (blue), deferoxamine (purple), mianserin (orange), dopamine (yellow), octopamine (dark purple). Scale bar, 1 mm. n = 11 (control), 13 (serotonin), 15 (deferoxamine), 17 (mianserin), 16 (dopamine), 12 (octopamine) biologically independent animals. p-value = 0.517 (serotonin vs. control), 0.377 (deferoxamine vs. control), 3.40e-08 (mianserin vs. control), 0.000354 (dopamine vs. control), 0.0540 (octopamine vs. control). Images (**c**) and quantification (**d**) of *fmo-2p*::*mCherry* exposed to water (pink), DR (blue), mianserin (purple) or both (orange) in combination with serotonin. Scale bar, 1 mm. n = 14 (fed), 12 (fed + mianserin), 13 (DR), 11 (DR + mianserin), 12 (fed + serotonin), 12 (DR + serotonin), 15 (fed + mianserin + serotonin), 9 (DR + mianserin +serotonin) biologically independent animals. p-value = 0.6315684 (fed vs. fed + serotonin), 0.06077056 (DR vs. DR + serotonin), 0.3074768 (fed + mianserin vs. fed + mianserin + serotonin), 0.1582417 (DR + mianserin vs. DR + mianserin + serotonin). Additional control images (**e**) and quantification (**f**) from Fig 2a-c. Scale bar, 1 mm. Images (**g**) quantified in Figure 2d. Scale bar, 1 mm. Images (**h**) quantified in Figure 2e. Scale bar, 1 mm. n = 16 (control fed), 19 (control fed + smell), 11 (control DR), 16 (control DR + smell), 14 (mianserin fed), 16 (mianserin fed + smell), 12 (mianserin DR), 13 (mianserin DR + smell), 11 (thioridazine fed), 10 (thioridazine fed + smell), 8 (thioridazine DR), 13 (thioridazine DR + smell) biologically independent animals. p-value = 0.412 (control fed vs. control fed + smell), 4.90e-08 (control fed vs. control DR), 5.53e-07 (control fed vs. control DR + smell), 6.04e-07 (mianserin fed vs. mianserin fed + smell), 4.99e-08 (mianserin fed vs. mianserin DR), 1.28e-08 (mianserin fed vs. mianserin DR + smell), 0.884 (thioridazine fed vs. thioridazine fed + smell), 0.00639 (thioridazine fed vs. thioridazine DR), 0.0541 (thioridazine fed vs. thioridazine DR + smell). qPCR results (**i**) for *fmo-2* mRNA levels after 8 hours post DR (orange), mianserin (purple) or thioridazine (blue) treatment normalized to water control. n = 3 (fed), 3 (+ thioridazine), 3 (+ mianserin), 3 (DR) biologically independent experiments. p-value = 0.0466 (thioridazine vs. fed), 0.0311 (mianserin vs. fed), 0.0314 (DR vs. fed). Images (**j**) and quantification (**k**) of *fmo-2p*::*mCherry* given 25µM of mianserin (blue) or thioridazine (purple) for indicated time points. Scale bar, 1 mm. n= 13 (fed none), 23 (fed mianserin 24 h), 9 (fed thioridazine 24 h) biologically independent animals. p-value = 4.40e-15 (fed none vs. fed mianserin 24 h), 0.0604 (fed none vs. fed thioridazine 24 h). n = 18 (fed none), 26 (fed mianserin 48 h), 23 (fed thioridazine 48 h) biologically independent animals. p-value = 1.17e-14 (fed none vs. fed mianserin 48 h), 0.0128 (fed none vs. fed thioridazine 48 h). n = 22 (fed none), 33 (fed mianserin 72 h), 42 (fed thioridazine 72 h) biologically independent animals. p-value = 1.16e-17 (fed none vs. fed mianserin 72 h), 7.59e-06 (fed none vs. fed thioridazine 72 h). n = 19 (fed none), 25 (fed mianserin 96 h), 25 (fed thioridazine 96 h) biologically independent animals. p-value = 1.61e-13 (fed none vs. fed mianserin 96 h), 3.54e-10 (fed none vs. fed thioridazine 96 h). Survival curves (**l**) of WT animals on fed conditions in pink and DR conditions in blue on water (solid lines) or 50µM mianserin (dotted lines). * denotes P<.05, ** denotes P<.01, *** denotes P<.001 and **** denotes P<.0001 when compared to fed (Welch Two Sample t-test, two-sided). The box plots display the median by the middle line of the box. The upper boundary of the box indicates the 75% interquartile range, and the lower boundary indicates the 25% interquartile range.

**
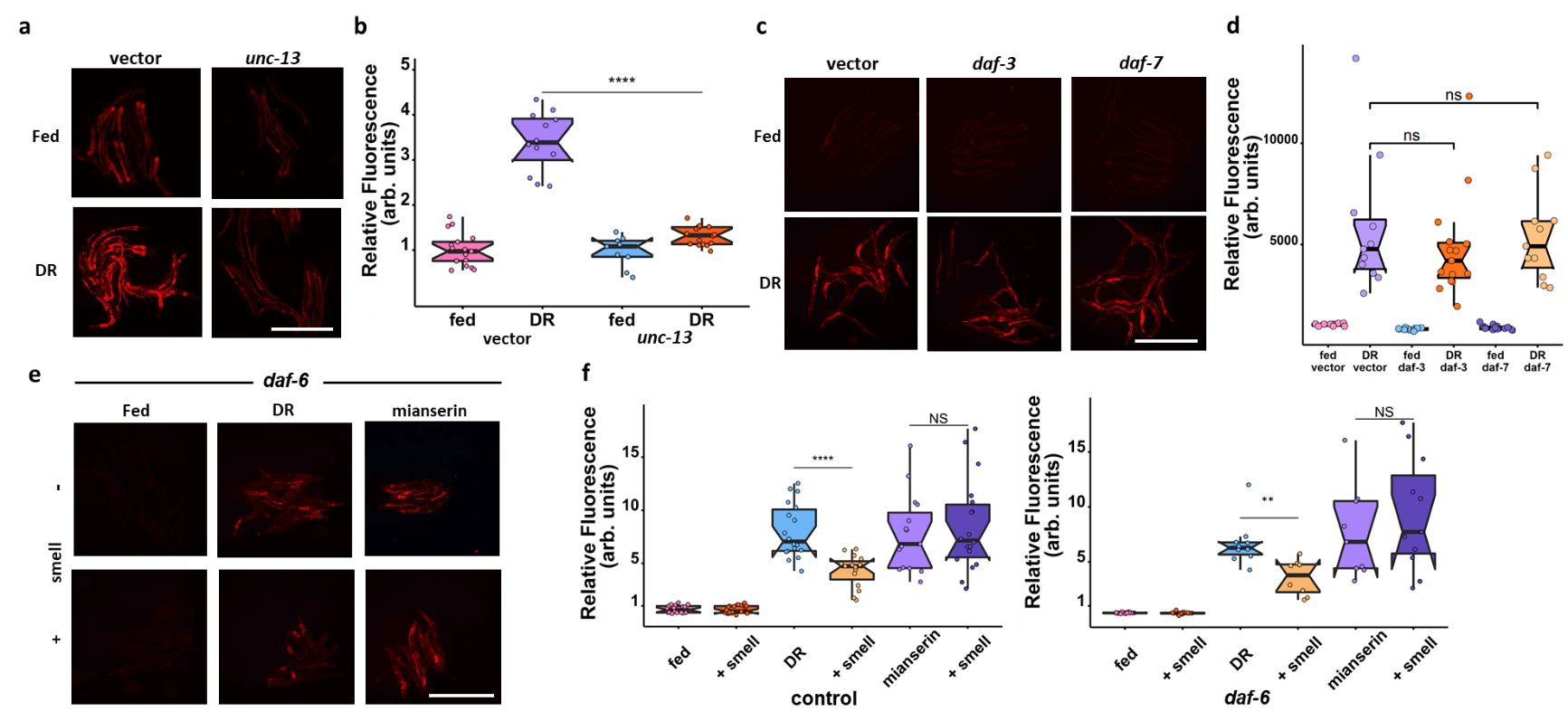
**

**
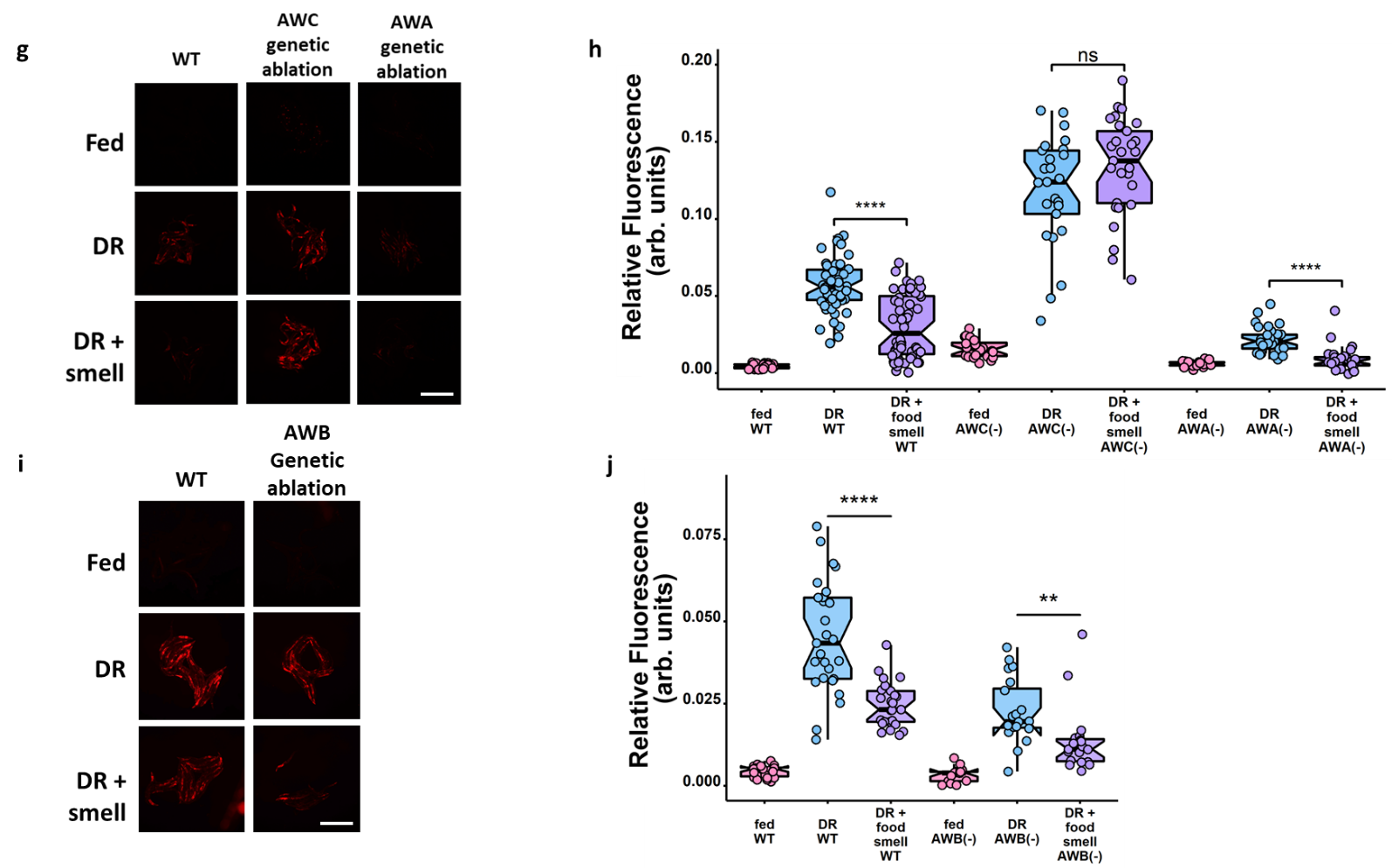
**

**Supplementary Fig. 4. Neuronal gene necessity for *fmo-2* induction under DR/food smell/biogenic amin antagonism.** Images (**a**) and quantification (**b**) of individual *fmo-2p::mCherry*; uIs60 [unc-119p::YFP + unc-119p::sid-1] (a neuronal RNAi hyperresponsive strain) worms on fed (pink, blue) and DR (purple, orange) fed on vector or *unc-13*  RNAi, respectively. Scale bar, 1 mm. n= 16 (vector fed), 12 (vector DR), 9 (*unc-13* fed), 15 (*unc-13* DR) biologically independent animals. p-value = 5.20e-07 (vector DR vs. *unc-13* DR). Images (**c**) and quantification (**d**) of individual *fmo-2p::mCherry* worms on vector, *daf-3*, and *daf-7* RNAi on fed or DR. Scale bar, 1 mm. n = 10 (fed vector), 11 (DR vector), 13 (fed *daf-3*), 15 (DR *daf-3*), 14 (fed *daf-7*), 11 (DR *daf-7*) biologically independent animals. p-value = 0.426 (DR vector vs. DR *daf-3*), 0.725 (DR vector vs. DR *daf-7*). Images (**e**) and quantification (**f**) of *fmo-2p*::*mCherry* in a *daf-6* KO background on fed, DR or exposed to mianserin and food smell. Scale bar, 1 mm. n = 16 (control fed), 17 (control fed + smell), 18 (control DR), 15 (control DR + smell), 14 (control mianserin), 17 (control mianserin + smell), 8 (*daf-6* fed), 9 (*daf-6* fed + smell), 10 (*daf-6* DR), 8 (*daf-6* DR + smell), 9 (*daf-6* mianserin), 11 (*daf-6* mianserin + smell) biologically independent animals. p-value = 0.000112 (control DR vs. control DR + smell), 0.528 (control mianserin vs. control mianserin + smell), 0.00330 (*daf-6* DR vs. *daf-6* DR + smell), 0.435 (*daf-6* mianserin vs. *daf-6* mianserin + smell). Images (**g**) and quantification (**h**) of *fmo-2p*::*mCherry* in a WT (control), AWC or AWA genetic ablation background on fed (pink) ,DR (blue) or DR exposed to food smell (purple). Scale bar, 1 mm. n = 24 (fed WT), 50 (DR WT), 56 (DR + food smell WT), 23 (fed AWC(-)), 25 (DR AWC(-)), 29 (DR + food smell AWC(-)), 20 (fed AWA(-)), 35 (DR AWA(-)), 28 (DR + food smell AWWA(-)) biologically independent animals. p-value = 7.78e-11 (WT DR vs. WT DR + food smell), 0.0979 (AWC(-) DR vs. AWC(-) DR + food smell), 9.94e-08 (AWA(-) DR vs. AWA(-) DR + food smell). Images (**i**) and quantification (**j**) of *fmo-2p*::*mCherry* in a WT (control) or AWB genetic ablation background on fed (pink), DR (blue) or DR exposed to food smell (purple). Scale bar, 1 mm. n = 25 (fed WT), 27 (WT DR), 25 (WT DR + food smell), 16 (AWB(-) fed), 20 (AWB(-) DR), 19 (AWB(-) DR + food smell) biologically independent animals. p-value = 1.21e-06 (WT DR vs. WT DR + food smell), p-value = 0.00804 (AWB(-) DR vs AWB(-) DR + food smell). ** denotes P<.01, **** denotes P<.0001 when compared to DR (Welch Two Sample t-test, two-sided). The box plots display the median by the middle line of the box. The upper boundary of the box indicates the 75% interquartile range, and the lower boundary indicates the 25% interquartile range.


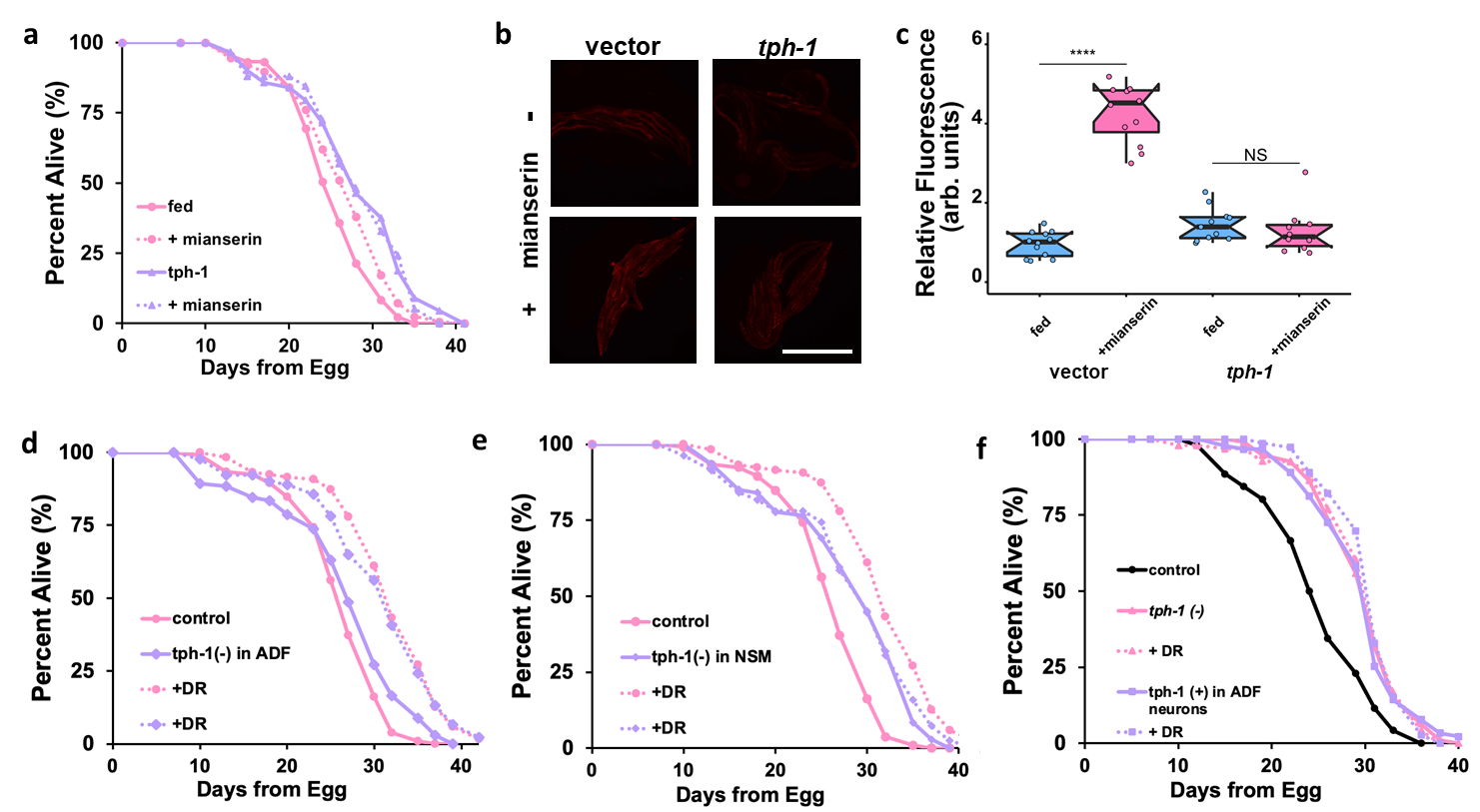


**
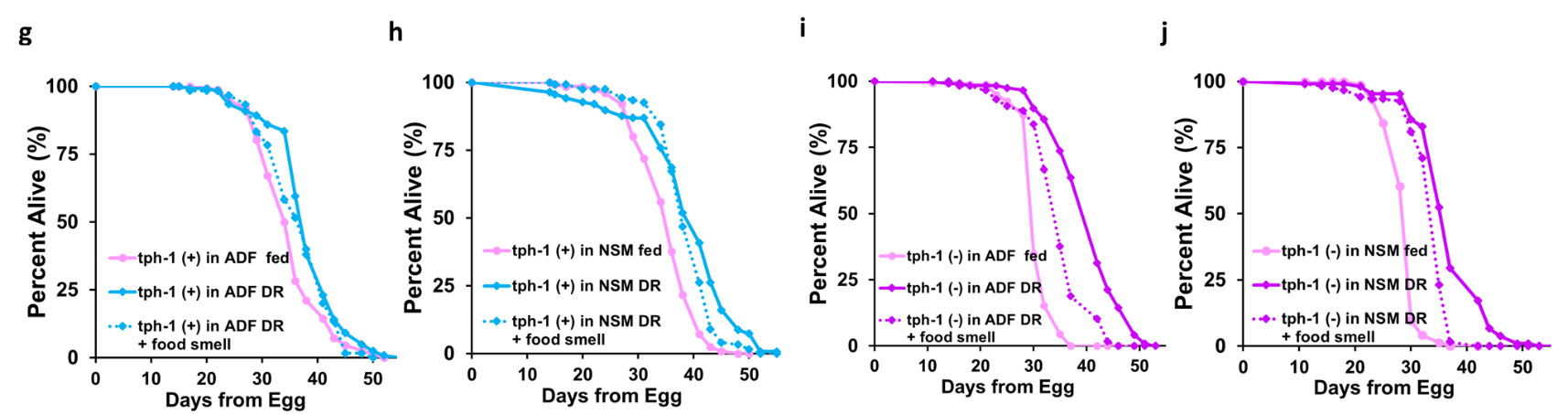
**

**Supplementary Fig. 5. Serotonin and serotonergic neuron-regulation of *fmo-2* induction and longevity.** Survival curves (**a**) of WT animals (pink) and *tph-1* KO animals (purple) on water (solid lines) or 50µM mianserin (dotted lines). Images (**b**) and quantification (**c**) of individual *fmo-2p::mCherry*; uIs60 [unc-119p::YFP + unc-119p::sid-1] (a neuronal RNAi hyperresponsive strain) worms on *tph-1* RNAi exposed to water (blue) or 50µM mianserin (pink) conditions. Scale bar, 1 mm. n = 12 (vector fed), 13 (vector + mianserin), 11 (*tph-1* fed), 10 (*tph-1* + mianserin) biologically independent animals. p-value = 1.51e-11 (vector fed vs. vector + mianserin), 0.478 (*tph-1* vector vs. *tph-1* + mianserin). Survival curves comparing control (pink) and *tph-1* ADF-specific KO (**d**) or NSM-specific KO (**e**) (purple) animals on fed (solid line) and DR (dotted lines). Control (pink) and DR (dotted pink line) survival curves in **d** and **e** are identical. Data in **d** and **e** were acquired concurrently. Survival curves (**f**) comparing control (black), *tph-1* KO (pink), and *tph-1* ADF-specific rescue (purple) animals on fed (solid line) and DR (dotted lines). Survival curves comparing *tph-1* ADF-specific rescue (**g**) or NSM-specific rescue (**h**) animals on fed (pink solid line), DR (blue solid line) or DR exposed to food smell (blue dotted line). Survival curves comparing *tph-1* ADF-specific KO (**i**) or NSM-specific KO (**j**) animals on fed (pink solid line), DR (purple solid line), or DR exposed to food smell (purple dotted line). **** denotes P<.0001 compared to fed (Welch Two Sample t-test, two-sided). The box plots display the median by the middle line of the box. The upper boundary of the box indicates the 75% interquartile range, and the lower boundary indicates the 25% interquartile range.

**
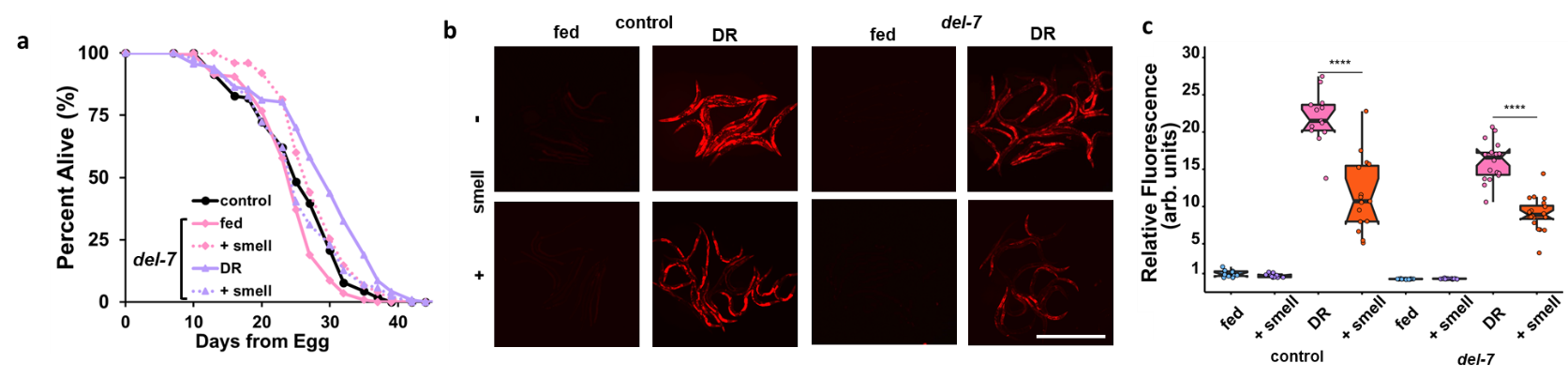
**

**
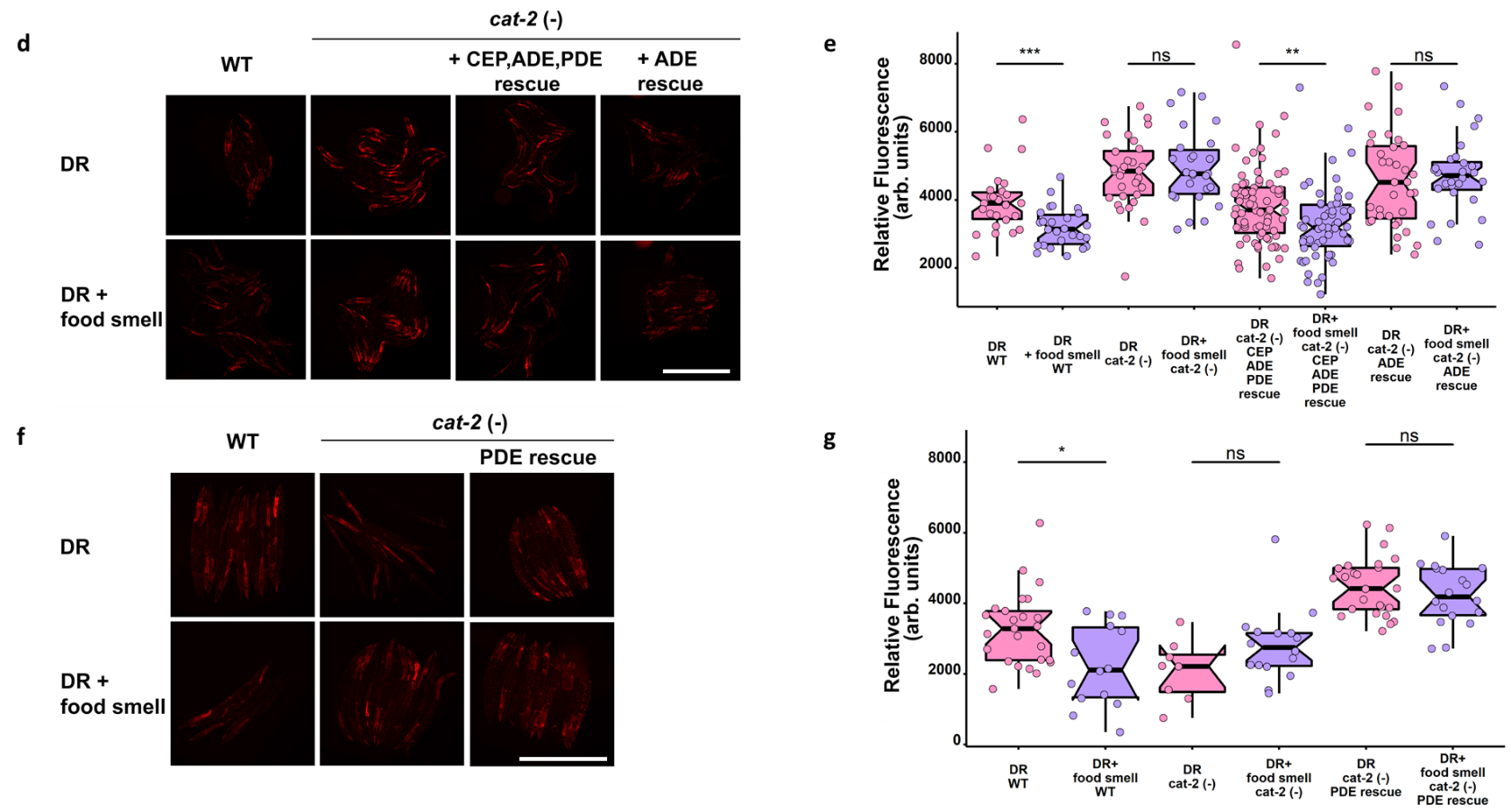
**

**Supplementary Fig. 6. *del-7* mutants look phenotypically wild type in their induction of *fmo-2* and lifespan extension; Dopaminergic ADE and PDE neurons are not involved in food odor signaling.** Survival curves (**a**) of conditions comparing WT (black) to *del-7* on fed (pink) and DR (purple) conditions in combination with food smell (dotted lines). Images (**b**) and quantification (**c**) of *fmo-2p*::*mCherry* in a WT (control) and *del-7* background on fed (blue) and DR (pink) exposed to food smell (purple and orange, respectively). Scale bar, 1 mm. n = 11 (control fed), 13 (control fed + smell), 13 (control DR), 15 (control DR + smell), 13 (*del-7* fed), 14 (*del-7* fed + smell), 18 (*del-7* DR), 20 (*del-7* DR + smell) biologically independent animals. p-value = 1.32e-06 (control DR vs. control DR + smell), 1.08e-10 (*del-7* DR vs. *del-7* DR + smell). Images (**d**) and quantification (**e**) of *fmo-2*p::*mCherry* in a WT, *cat-2* KO, *cat-2* KO with *cat-2* CEP, ADE, PDE neurons rescue or *cat-2* KO with *cat-2* ADE neuron rescue on DR (pink) and DR + food smell (purple). Scale bar, 1 mm. n = 26 (DR WT), 26 (DR + food smell WT), 32 (DR *cat-2*(-)), 27 (DR + food smell *cat-2*(-)), 73 (DR *cat-2*(-) CEP ADE PDE rescue), 58 (DR + food smell *cat-2*(-) CEP ADE PDE rescue), 35 (DR cat-2(-) ADE rescue), 28 (DR + food smell *cat-2*(-) ADE rescue) biologically independent animals. p-value = 0.000711 (DR WT vs. DR + food smell WT), 0.613 (DR *cat-2*(-) vs. DR + food smell *cat-2*(-)), 0.00702 (DR *cat-2*(-) CEP ADE PDE rescue vs. DR + food smell *cat-2*(-) CEP ADE PDE rescue), 0.625 (DR *cat-2*(-) ADE rescue vs. DR + food smell *cat-2*(-) ADE rescue). Images (**f**) and quantification (**g**) of *fmo-2*p::*mCherry* in a WT, *cat-2* KO, or *cat-2* KO with *cat-2* PDE neuron rescue on DR (pink) and DR + food smell (purple). Scale bar, 1 mm. n = 25 (DR WT), 14 (DR + food smell WT), 8 (DR *cat-2*(-)), 16 (DR + food smell *cat-2*(-)), 25 (DR *cat-2*(-) PDE rescue), 18 (DR + food smell *cat-2*(-) PDE rescue) biologically independent animals. p-value = 0.0103 (DR WT vs. DR + food smell WT), 0.0922 (DR *cat-2*(-) vs. DR + food smell *cat-2*(-)), 0.347 (DR *cat-2*(-) PDE rescue vs. DR + food smell *cat-2*(-) PDE rescue). * denotes P<.05; ** denotes P<.01; *** denotes P<.001; **** denotes P<.0001 compared to DR (Welch Two Sample t-test, two-sided). The box plots display the median by the middle line of the box. The upper boundary of the box indicates the 75% interquartile range, and the lower boundary indicates the 25% interquartile range.


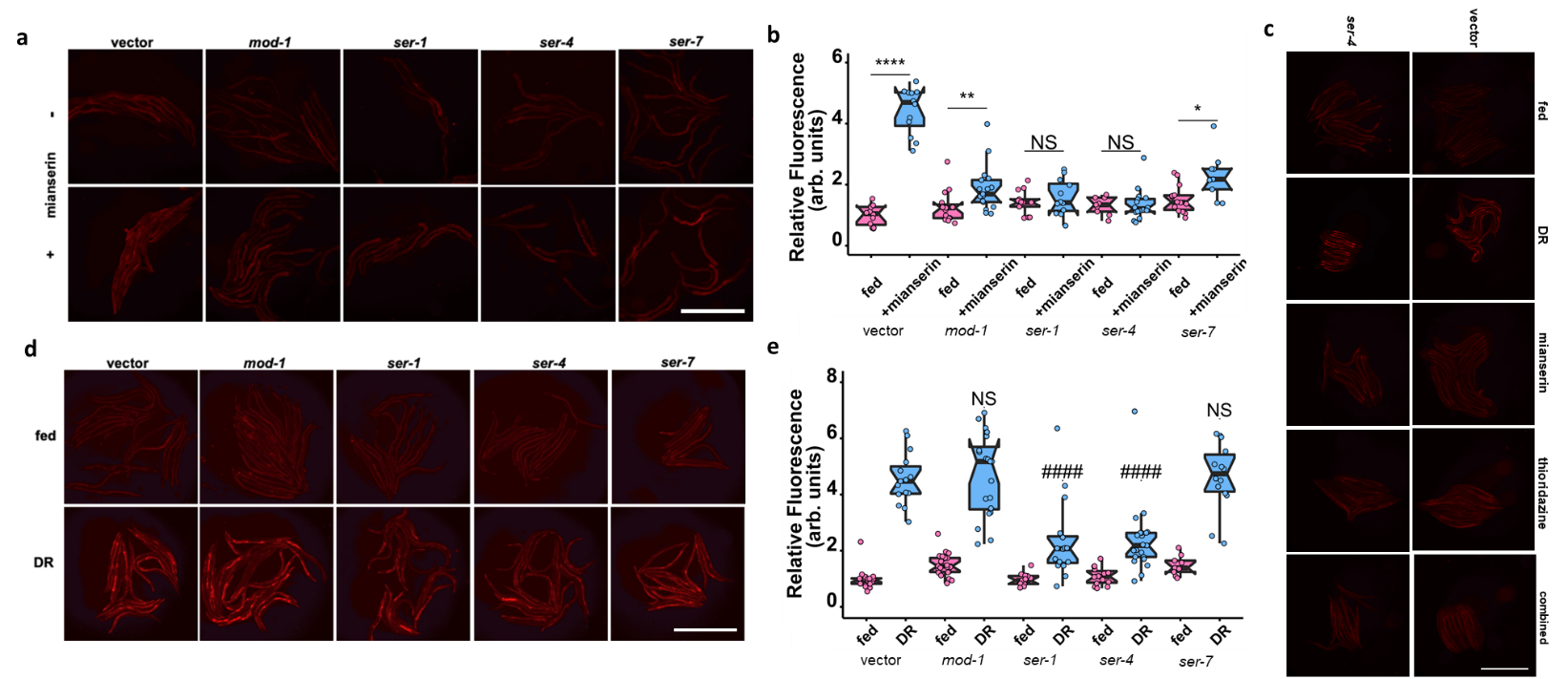


***
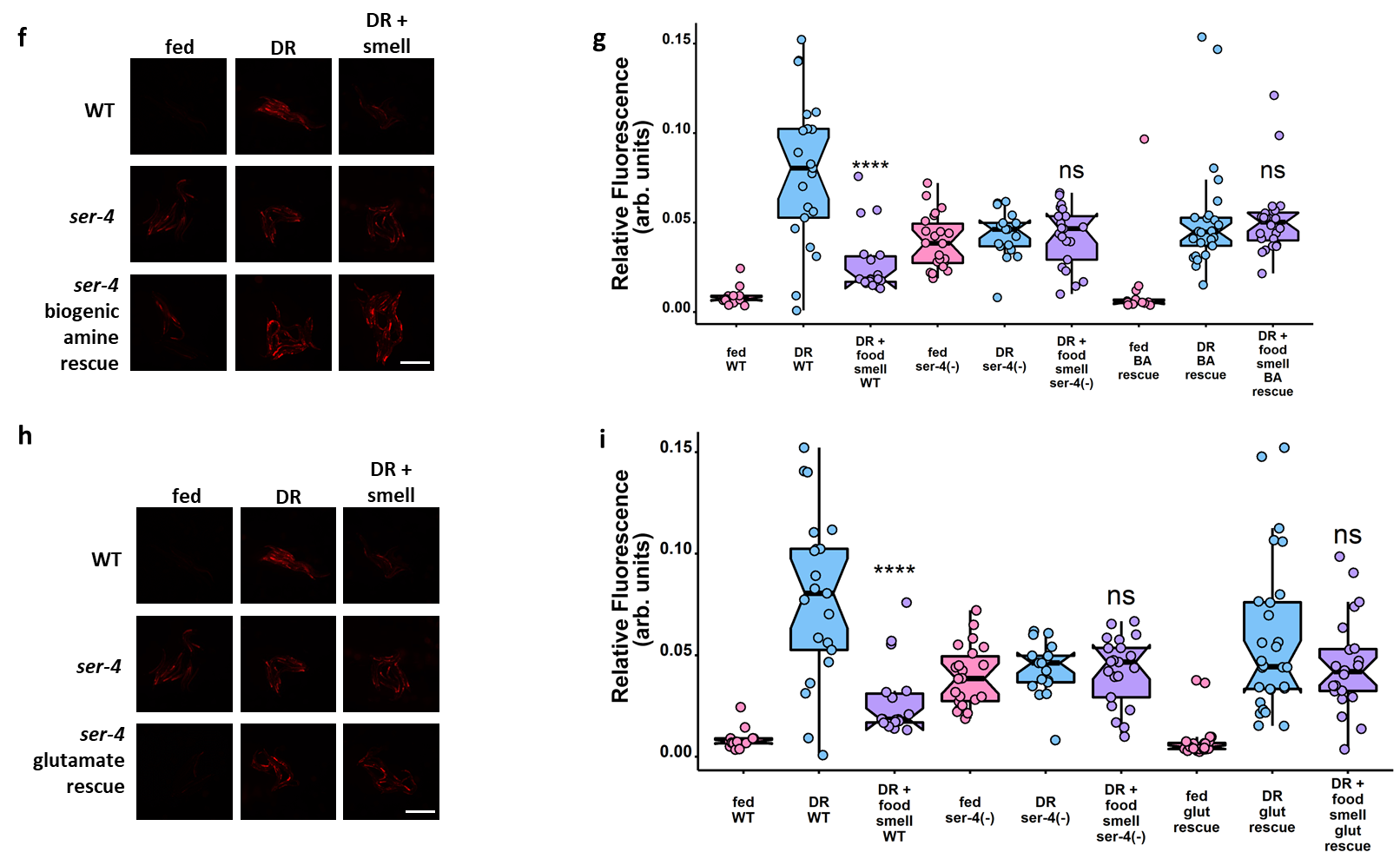
***

**Supplementary Fig. 7.** **The role of serotonergic receptor signaling in *fmo-2* induction by DR and DR mimetics.** Images (**a**) and quantification (**b**) of *fmo-2p*::*mCherry* grown on serotonin receptor RNAi exposed to water (pink) or 50µM mianserin (blue). Scale bar, 1 mm. n = 12 (vector fed), 13 (vector + mianserin), 15 (*mod-1* RNAi fed), 17 (*mod-1* RNAi + mianserin), 13 (*ser-1* RNAi fed), 12 (*ser-1* RNAi + mianserin), 9 (*ser-4* RNAi fed), 16 (*ser-4* RNAi + mianserin), 14 (*ser-7* RNAi fed), 9 (*ser-7* RNAi + mianserin) biologically independent animals. p-value = 2.15e-09 (vector fed vs. vector + mianserin), 0.0142 (*mod-1* RNAi fed vs. *mod-1* RNAi + mianserin), 0.450 (*ser-1* RNAi fed vs. *ser-1* RNAi + mianserin), 0.799 (*ser-4* RNAi fed vs. *ser-4* RNAi + mianserin), 0.0194 (*ser-7* RNAi fed vs. *ser-7* RNAi + mianserin). Images (**c**) quantified in Figure 4a. Images (**d**) and quantification (**e**) of *fmo-2p*::*mCherry* grown on serotonin receptor RNAi exposed to fed (pink) or DR (blue). Scale bar, 1 mm. n = 19 (vector fed), 15 (vector DR), 23 (*mod-1* RNAi fed), 20 (*mod-1* RNAi DR), 13 (*ser-1* RNAi fed), 16 (*ser-1* RNAi DR), 15 (*ser-4* RNAi fed), 21 (*ser-4* RNAi DR), 9 (*ser-7* RNAi fed), 14 (*ser-7* RNAi DR) biologically independent animals. p-value = 0.724 (vector DR vs. *mod-1* RNAi DR), 2.07e-05 (vector DR vs. *ser-1* RNAi DR), 1.55e-06 (vector DR vs. *ser-4* RNAi DR), 0.818 (vector DR vs. *ser-7* RNAi DR). * denotes P<.05, ** denotes P<.01, **** denotes P<.001 when compared to fed (Welch Two Sample t-test, two-sided). #### denotes P< .0001 when compared to DR (Welch Two Sample t-test, two-sided). Images (**f**) and quantification (**g**) of *fmo-2p*::*mCherry* or *ser-4* KO with *ser-4* biogenic amine rescue on fed (pink), DR (blue) or DR exposed to food smell (purple). Scale bar, 1 mm. n = 14 (WT fed), 21 (WR DR), 18 (WT DR = food smell), 22 (*ser-*4(-) fed), 17 (*ser-*4(-) DR), 21 (*ser-*4(-) DR + food smell), 13 (*ser-*4(-) BA rescue fed), 26 (*ser-*4(-) BA rescue DR), 24 (*ser-*4(-) BA rescue DR + food smell) biologically independent animals. p-value = 1.72e-05 (WT DR vs. WT DR + food smell), 0.849 (*ser-*4(-) DR vs. *ser-*4(-) DR + food smell), 0.927 (*ser-*4(-) BA rescue DR vs. *ser-*4(-) BA rescue DR + food smell). Images (**h**) and quantification (**i**) of *fmo-2p*::*mCherry* or *ser-4* KO with *ser-4* glutamatergic rescue on fed (pink), DR (blue) or DR exposed to food smell (purple). Scale bar, 1 mm. n = 14 (WT fed), 21 (WR DR), 18 (WT DR = food smell), 22 (*ser-*4(-) fed), 17 (*ser-*4(-) DR), 21 (*ser-*4(-) DR + food smell), 24 (*ser-*4(-) glut rescue fed), 28 (*ser-*4(-) glut rescue DR), 23 (*ser-*4(-) glut rescue DR + food smell) biologically independent animals. p-value = 1.72e-05 (WT DR vs. WT DR + food smell), 0.849 (*ser-*4(-) DR vs. *ser-*4(-) DR + food smell), 0.153 (*ser-*4(-) glut rescue DR vs. *ser-*4(-) glut rescue DR + food smell). **** denotes P<.0001 when compared to DR (Welch Two Sample t-test, two-sided). The box plots display the median by the middle line of the box. The upper boundary of the box indicates the 75% interquartile range, and the lower boundary indicates the 25% interquartile range.

**
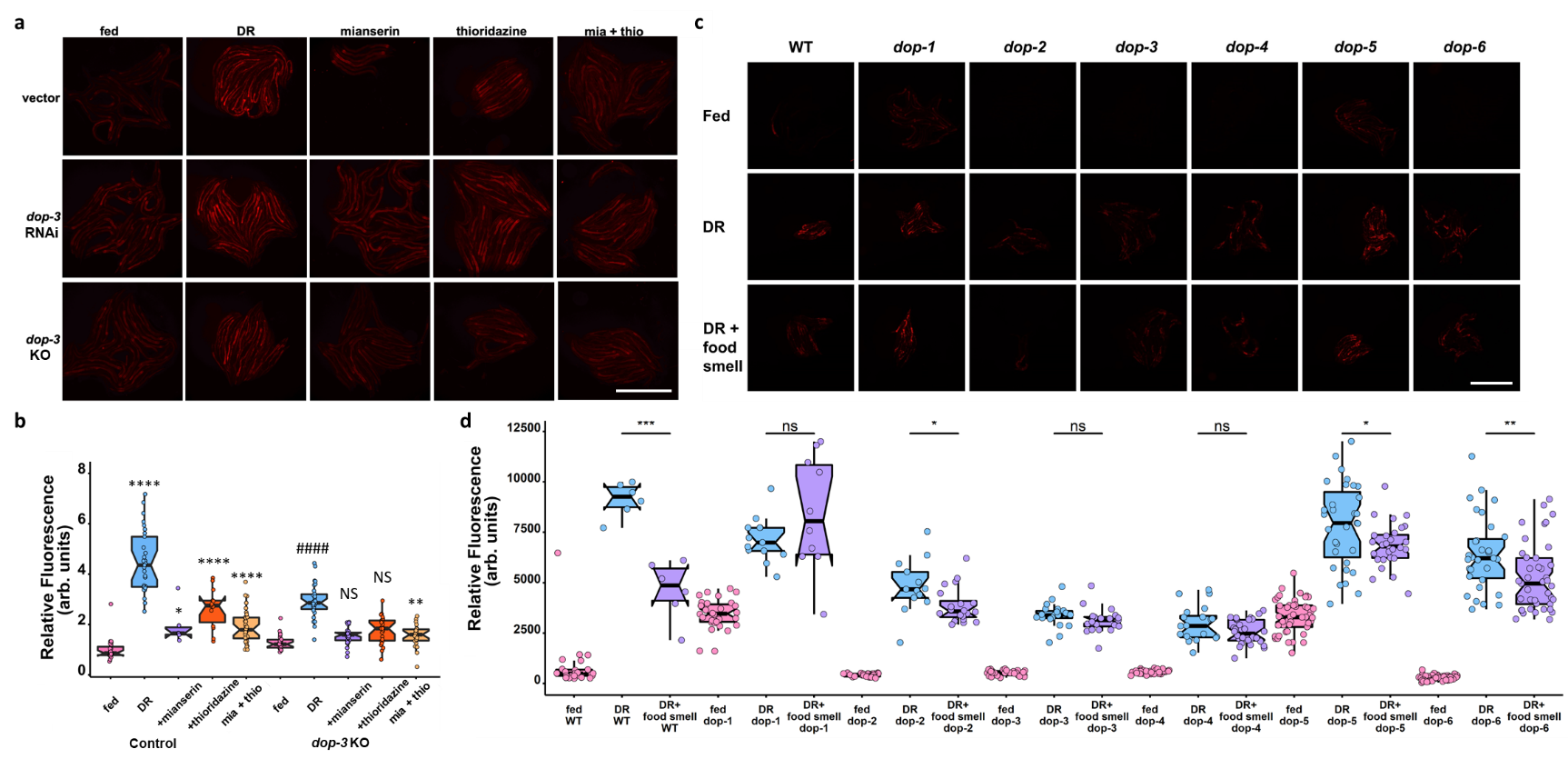
**

**
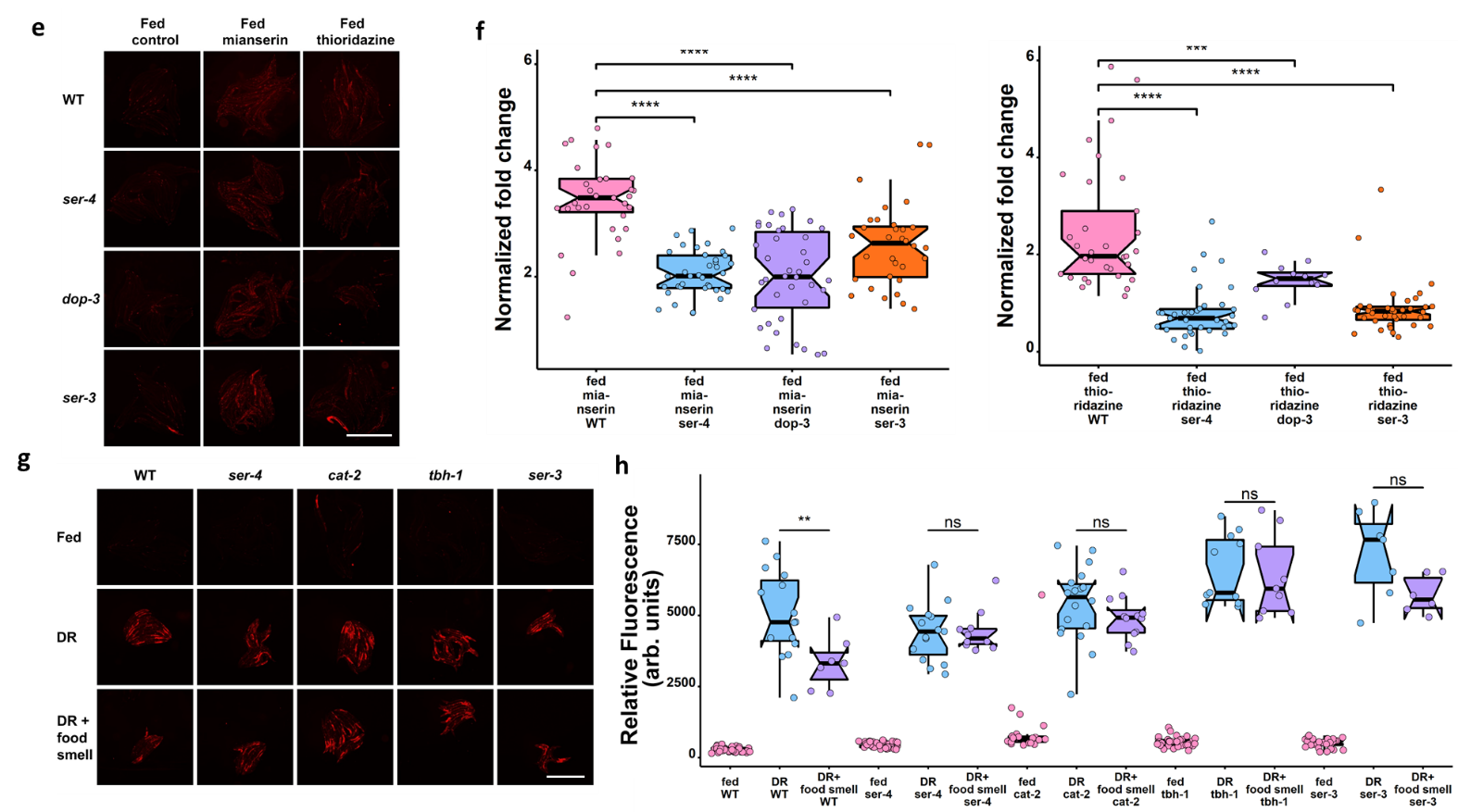
**

**Supplementary Fig. 8. The role of dopamine and octopamine signaling in *fmo-2* induction by DR and DR mimetics.** Images (**a**) quantified in Figure 4f. Images (**a**) and quantification (**b**) of WT *fmo-2p*::*mCherry* or *dop-3* KO on fed (pink), and DR (blue) treated with 100µM mianserin (purple), 100 µM thioridazine (orange), or combined (yellow). Scale bar, 1 mm. n = 23 (control fed), 29 (control DR), 6 (control + mianserin), 14 (control + thioridazine), 34 (control + mia + thio), 25 (*dop-3*KO fed), 23 (*dop-3*KO DR), 27 (*dop-3*KO + mianserin), 22 (*dop-3*KO + thioridazine), 21 (*dop-3*KO + mia + thio) biologically independent animals. p- value = 1.69e-16 (control fed vs. control DR), 0.0268 (control fed vs. control mianserin), 8.34e-07 (control fed vs. control thioridazine), 1.03e-07 (control fed vs. control mia + thio), 1.38e-08 (*dop-3*KO fed vs. *dop-3*KO DR), 0.565 (*dop-3*KO fed vs. *dop-3*KO + mianserin), 0.130 (*dop-3*KO fed vs. *dop-3*KO + thioridazine), 0.00406 (*dop-3*KO fed vs. *dop-3*KO mia + thio). Images (**c**) and quantification (**d**) of WT *fmo-2p*::*mCherry*, *dop-1*, *dop-2*, *dop-3*, *dop-4*, *dop-5*, or *dop-6* KO on fed (pink), DR (blue), or DR exposed to food smell (purple). Scale bar, 1 mm. n = 24 (fed WT), 6 (DR WT), 6 (DR + food smell WT), 32 (fed *dop-1*), 13 (DR *dop-1*), 10 (DR + food smell *dop-1*), 27 (fed *dop-2*), 13 (DR *dop-2*), 20 (DR + food smell *dop-2*), 29 (fed *dop-3*), 22 (DR *dop-3*), 21 (DR + food smell *dop-3*), 39 (fed *dop-4*), 20 (DR *dop-4*), 32 (DR + food smell *dop-4*), 49 (fed *dop-5*), 31 (DR *dop-5*), 28 (DR + food smell *dop-5*), 61 (fed *dop-6*), 31 (DR *dop-6*), 41 (DR + food smell *dop-6*) biologically independent animals. p-value = 0.000184 (DR WT vs. DR + food smell WT), 0.208 (DR *dop-1* vs. DR + food smell *dop-1*), 0.0344 (DR *dop-2* vs. DR + food smell *dop-2*), 0.169 (DR *dop-3* vs. DR + food smell *dop-3*), 0.0670 (DR *dop-4* vs. DR + food smell *dop-4*), 0.0261 (DR *dop-5* vs. DR + food smell *dop-5*), 0.00705 (DR *dop-6* vs. DR + food smell *dop-6*). Images (**e**) and *fmo-2* induction fold change (**f**) of WT *fmo-2p*::*mCherry*, *ser-4*, *dop-6,* or *ser-3* KO on fed treated with 100 µM mianserin or thioridazine normalized to water control. Scale bar, 1 mm. n = 31 (fed mianserin WT), 37 (fed mianserin *ser-4*), 38 (fed mianserin *dop-3*), 32 (fed mianserin *ser-3*), 33 (fed thioridazine WT), 35 (fed thioridazine *ser-4*), 12 (fed thioridazine *dop-3*), 19 (fed thioridazine *ser-3*) biologically independent animals. p-value = 1.57e-11 (fed mianserin WT vs. fed mianserin *ser-4*), 4.89e-10 (fed mianserin WT vs. fed mianserin *dop-3*), 6.13e-05 (fed mianserin WT vs. fed mianserin *ser-3*), 1.00e-08 (fed thioridazine WT vs. fed thioridazine *ser-4*), 0.000150 (fed thioridazine WT vs. fed thioridazine *dop-3*), 3.02e-08 (fed thioridazine WT vs. fed thioridazine *ser-3*). Images (**g**) and quantification (**h**) of WT *fmo-2p*::*mCherry*, *ser-4*, *cat-2*, *tbh-1*, or *ser-3* KO on fed (pink), DR (blue), or DR exposed to food smell (purple). Scale bar, 1 mm. n = 36 (fed WT), 15 (DR WT), 7 (DR + food smell WT), 36 (fed *ser-4*), 15 (DR *ser-4*), 10 (DR + food smell *ser-4*), 22 (fed *cat-2*), 18 (DR *cat-2*), 12 (DR + food smell *cat-2*), 35 (fed *tbh-1*), 11 (DR *tbh-1*), 9 (DR + food smell *tbh-1*), 33 (fed *ser-3*), 7 (DR *ser-3*), 6 (DR + food smell *ser-3*) biologically independent animals. p-value = 0.00430 (DR WT vs. DR + food smell WT), 0.928 (DR *ser-4* vs. DR + food smell *ser-4*), 0.228 (DR *cat-2* vs. DR + food smell *cat-2*), 0.763 (DR *tbh-1* vs. DR + food smell *tbh-1*), 0.0547 (DR *ser-3* vs. DR + food smell *ser-3*). * denotes P<.05, ** denotes P<.01, *** denotes P<.001 and **** denotes P<.0001 when compared to fed (Welch Two Sample t-test, two-sided). #### denotes P<.0001 when compared to control DR (Welch Two Sample t-test, two-sided). The box plots display the median by the middle line of the box. The upper boundary of the box indicates the 75% interquartile range, and the lower boundary indicates the 25% interquartile range.

**
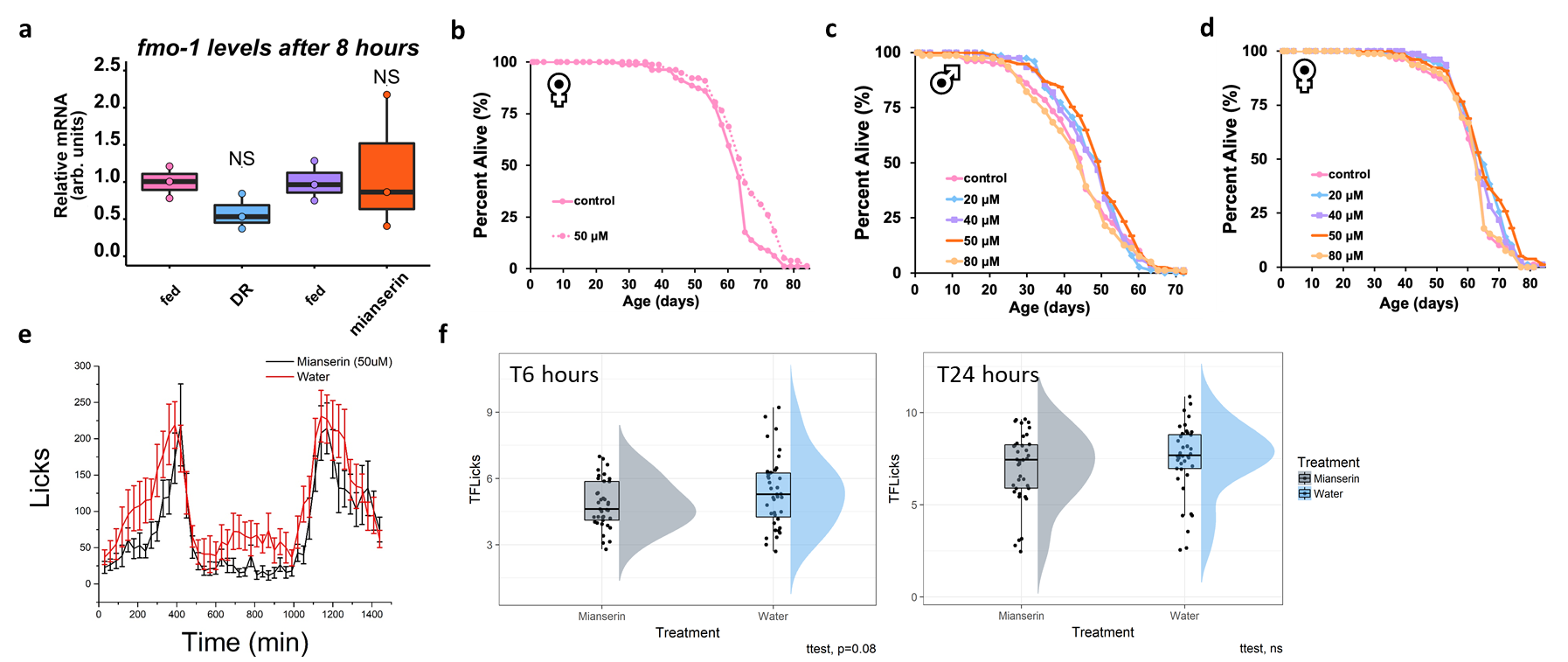
**

**Supplementary Fig. 9. Effects of DR mimetic mianserin on Fmo expression, feeding, and lifespan.** Fmo-1 mRNA levels (**a**) after 8 hours of starvation (blue) or 100µM mianserin (orange) compared to water controls (pink and purple, respectively). n = 3 (fed), 3 (DR), 3 (fed for mianserin), 3 (mianserin) biologically independent experiments. p-value = 0.0893 (fed vs. DR), 0.809 (fed vs. mianserin) (Welch Two Sample t-test, two-sided). Survival curves of female (**b**) flies dosed with water (solid line) or 50µM (dotted line) mianserin. Combined survival curves of male (**c**) and female (**d**) flies dosed with water (pink), 20µM (blue), 40µM (purple), 50µM (orange), or 80µM (yellow) mianserin. 24 hours of FLIC assay data monitoring food intake in control (black) and mianserin (red) treatment (**e**). Extracted FLIC data (**f**) at 6 and 24 hours. Results are displayed as mean ± SEM in **e**. n = 32 (Water), 32 (Mianserin) biologically independent animals. The box plots display the median by the middle line of the box. The upper boundary of the box indicates the 75% interquartile range, and the lower boundary indicates the 25% interquartile range.


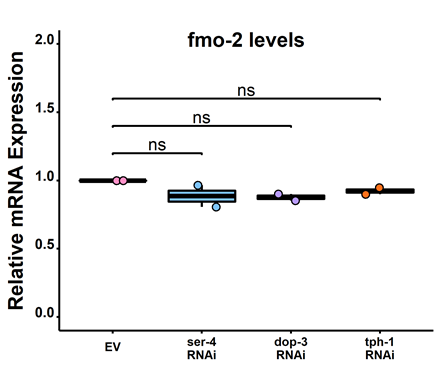


**Supplementary Fig. 10. Fmo-2 levels of *ser-4*, *dop-3*, or *tph-1* knockdown.** Fmo-2 mRNA levels of worms fed on vector, *ser-4*, *dop-3*, or *tph-1* RNAi. n = 2 (EV), 2 (*ser-4* RNAi), 2 (*dop-3* RNAi), 2 (*tph-1* RNAi) biologically independent experiments. p-value = 0.387 (EV vs. *ser-4* RNAi), 0.124 (EV vs. *dop-3* RNAi), 0.194 (EV vs. *tph-1* RNAi) (Welch Two Sample t-test, two-sided).

**References:**

1. Fitch DHA. Introduction to nematode evolution and ecology. WormBook, ed. The C.

elegans Research Community: Wormbook; 2005.

2. Frokjaer-Jensen C, Wayne Davis M, Hopkins CE, et al. Single-copy insertion of transgenes in Caenorhabditis elegans. 10.1038/ng.248. *Nat Genet*. 2008;40(11):1375-1383. doi:<http://www.nature.com/ng/journal/v40/n11/suppinfo/ng.248_S1.html>

3. Berkowitz LA, Knight AL, Caldwell GA, Caldwell KA. Generation of stable transgenic C. elegans using microinjection. *J Vis Exp*. Aug 2008;(18)doi:10.3791/833

4. Sutphin GL, Kaeberlein M. Measuring Caenorhabditis elegans life span on solid media. *J Vis Exp*. 2009;(27)doi:1152 [pii]

10.3791/1152

5. Anderson EN, Corkins ME, Li JC, et al. C. elegans lifespan extension by osmotic stress requires FUdR, base excision repair, FOXO, and sirtuins. M*ech Ageing Dev.* Mar 2016;154:30-42. doi:10.1016/j.mad.2016.01.004

6. Maier W, Adilov B, Regenass M, Alcedo J. A neuromedin U receptor acts with the sensory system to modulate food type-dependent effects on C. elegans lifespan. P*LoS Biol.* May 25 2010;8(5):e1000376. doi:10.1371/journal.pbio.1000376

7. Pang S, Curran SP. Adaptive capacity to bacterial diet modulates aging in C. elegans. C*ell Metab.* Feb 04 2014;19(2):221-31. doi:10.1016/j.cmet.2013.12.005

8. Petrascheck M, Ye X, Buck LB. A high-throughput screen for chemicals that increase the lifespan of Caenorhabditis elegans. A*nn N Y Acad Sci.* Jul 2009;1170:698-701. doi:10.1111/j.1749-6632.2009.04377.x

9. Zarse K, Ristow M. Antidepressants of the serotonin-antagonist type increase body fat and decrease lifespan of adult Caenorhabditis elegans. P*LoS One.* 2008;3(12):e4062. doi:10.1371/journal.pone.0004062

10. Greer EL, Brunet A. Different dietary restriction regimens extend lifespan by both independent and overlapping genetic pathways in C. elegans. A*ging Cell.* Apr 2009;8(2):113-27. doi:10.1111/j.1474-9726.2009.00459.x

11. Bargmann CI, Hartwieg E, Horvitz HR. Odorant-selective genes and neurons mediate olfaction in C. elegans. C*ell.* Aug 1993;74(3):515-27. doi:10.1016/0092-8674(93)80053-h

12. Worthy SE, Haynes L, Chambers M, et al. Identification of attractive odorants released by preferred bacterial food found in the natural habitats of C. elegans. P*LoS One.* 2018;13(7):e0201158. doi:10.1371/journal.pone.0201158

13. Linford NJ, Bilgir C, Ro J, Pletcher SD. Measurement of lifespan in Drosophila melanogaster. J *Vis Exp.* Jan 2013;(71)doi:10.3791/50068

14. Murakami S, Salmon A, Miller RA. Multiplex stress resistance in cells from long-lived dwarf mice. F*ASEB J.* Aug 2003;17(11):1565-6. doi:10.1096/fj.02-1092fje

15. Han SK, Lee D, Lee H, et al. OASIS 2: online application for survival analysis 2 with features for the analysis of maximal lifespan and healthspan in aging research. O*ncotarget.* Aug 30 2016;7(35):56147-56152. doi:10.18632/oncotarget.11269
