# Supplementary material for: Serotonin and dopamine modulate aging in response to food odor and availability
Source: Nat Commun. 2022 Jun 7;13:3271. doi: 10.1038/s41467-022-30869-5 (PMC9174215; doi:10.1038/s41467-022-30869-5)
Supplement: Supplementary file 3 — Description of Additional Supplementary Files [file 41467_2022_30869_MOESM3_ESM.pdf]

Description of Additional Supplementary Files

File name: Supplementary Data 1.

Description: *C. elegans* and RNAi strains used in this study. All strains and RNAi conditions used in this study are listed.

File name: Supplementary Data 2.

Description: lifespan statistics. All lifespan replicates in this study are listed. Statistical summary of lifespans, statistics of main figure panels replicates, and supplementary Figure panels replicates are listed.

File name: Supplementary Data 3.

Description: Dosing and preparation of odorants. Summary of odorants preparation and dosing, and summary of odorants dosing and *fmo-2* induction under DR and fed are listed.
